# Supplementary material for: Hybrid Models and Biological Model Reduction with PyDSTool
Source: PLoS Comput Biol. 2012 Aug 9;8(8):e1002628. doi: 10.1371/journal.pcbi.1002628 (PMC3415397; doi:10.1371/journal.pcbi.1002628)
Supplement: Text S4 — Complete source code for the PyDSTool package (version 0.88.120504). Includes API documentation and help files linking to web pages. This file is identical to the current public release on Sourceforge.net. (ZIP) [file pcbi.1002628.s004.zip › PyDSTool/html/toc-PyDSTool.errors-module.html]

xml version="1.0" encoding="ascii"?


errors


# Module errors

---

## Classes

PyDSTool\_AttributeError  
 PyDSTool\_BoundsError  
 PyDSTool\_ClearError  
 PyDSTool\_ContError  
 PyDSTool\_Error  
 PyDSTool\_ExistError  
 PyDSTool\_InitError  
 PyDSTool\_KeyError  
 PyDSTool\_TypeError  
 PyDSTool\_UncertainValueError  
 PyDSTool\_UndefinedError  
 PyDSTool\_ValueError  

---

[hide private]
